# Supplementary material for: High Oxygen Shocking Reduces Postharvest Disease and Maintains Satisfying Quality in Fresh Goji Berries during Cold Storage by Affecting Fungi Community Composition
Source: Foods. 2023 Jun 29;12(13):2548. doi: 10.3390/foods12132548 (PMC10341370; doi:10.3390/foods12132548)
Supplement: Supplementary file 1 [file foods-12-02548-s001.zip › foods-2448709-supplementary.pdf]

Supplementary Figures

Figure S1

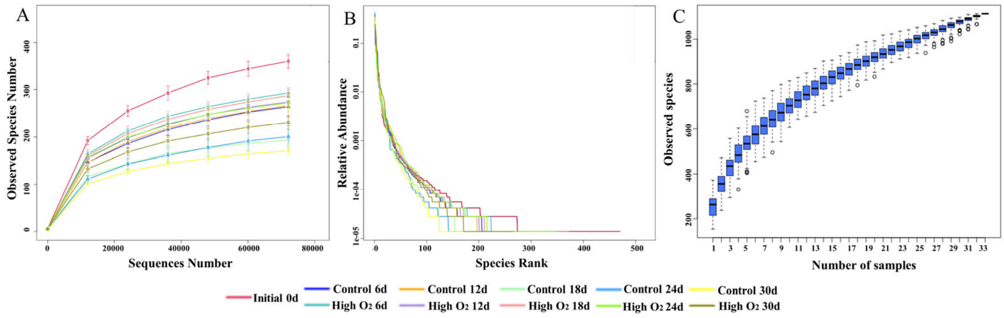

Figure S1. The fungal rarefaction curve, rank abundance, and species accumulation boxplot.

Figure S2

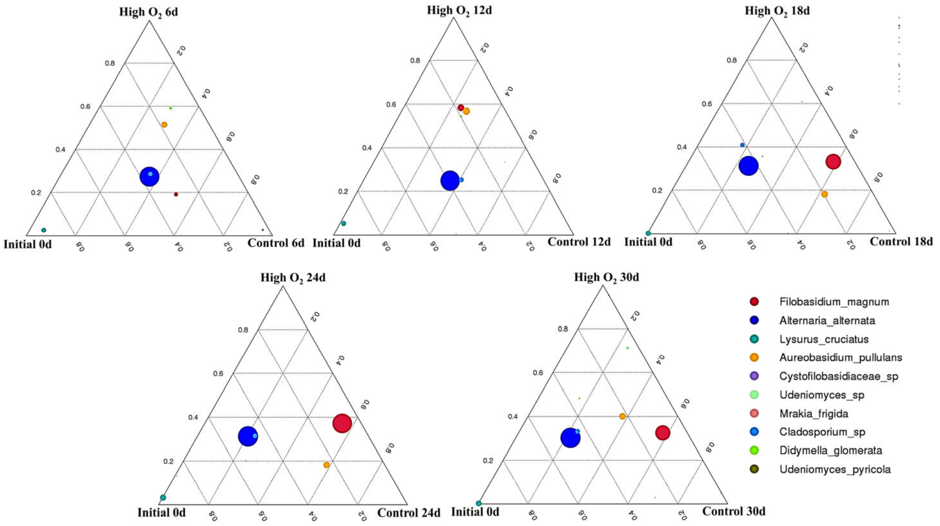

Figure S2. Ternary plot plotted in R software (Version 2.15.3), the size of the circle indicates the abundance information of different species. The closer of the circle to the vertex represents the higher content of this species in this group.

Supplementary Table

Table S1 The relative abundance of the dominant genera （top 10-group） of goji berries at 0 ± 0.5 °C storage.

Samples	*Filobasidium*	*Alternaria*	*Lysurus*	*Aureobasidium*	*Unidentified*	*Udeniomyces*	*Mrakia*	*Cladosporium*	*Didymella*	*Vishniacozyma*	*Others*
Initial_0d	0.065681	0.391111	0.218097	0.056145	0.002067	0.00536	0.000032	0.073417	0.017064	0.000398	0.170627
Control_6d	0.107444	0.390667	0.013836	0.09506	0.076409	0.003427	0.003898	0.075115	0.040922	0.00099	0.192233
High O_2_6d	0.04106	0.294363	0.006224	0.157369	0.00228	0.008953	0.000028	0.059979	0.08182	0.000874	0.347049
Control_12d	0.082814	0.362347	0.003417	0.092368	0.00215	0.020246	0.00006	0.087767	0.021892	0.00221	0.324727
High O_2_12d	0.202152	0.250316	0.012689	0.193542	0.000023	0.013568	0	0.054443	0.044579	0.002206	0.226481
Control_18d	0.411713	0.225848	0.000606	0.177412	0.002701	0.018724	0.001582	0.031673	0.013092	0.004564	0.112087
High O_2_18d	0.239994	0.281198	0.000523	0.051766	0.000014	0.037453	0.000023	0.073459	0.016722	0.003353	0.295496
Control_24d	0.438137	0.165134	0.000398	0.139843	0.000023	0.006895	0.000009	0.037689	0.007455	0.051271	0.153147
High O_2_24d	0.293878	0.256175	0.007445	0.043784	0.000314	0.034457	0.002178	0.050883	0.00615	0.004555	0.300181
Control_30d	0.359753	0.179284	0.000287	0.095652	0.005193	0.026512	0.044441	0.039261	0.007695	0.018257	0.223665
High O_2_30d	0.208525	0.246944	0.000532	0.101196	0.000217	0.109997	0.002308	0.055937	0.022142	0.022724	0.229478
